# FAM129B is a novel regulator of Wnt/β-catenin signal transduction in melanoma cells

**DOI:** 10.12688/f1000research.2-134.v2

**Published:** 2013-10-10

**Authors:** Willliam Conrad, Michael B Major, Michele A Cleary, Marc Ferrer, Brian Roberts, Shane Marine, Namjin Chung, William T Arthur, Randall T Moon, Jason D Berndt, Andy J Chien

**Affiliations:** 1 The Howard Hughes Medical Institute, Seattle WA, 98195, USA; 2 Department of Pharmacology, University of Washington School of Medicine, Seattle WA, 98195, USA; 3 Department of Cell Biology and Physiology, UNC Lineberger Comprehensive Cancer Center, University of North Carolina at Chapel Hill, Chapel Hill NC, 27599-7295, USA; 4 Rosetta Inpharmatics, LLC, Merck & Co Inc., Seattle WA, 98109, USA; 5 Merck & Co., Inc., West Point PA, 19486, USA; 6 Department of Automated Biotechnology, Merck, North Wales PA, 19454, USA; 7 NCATS/NIH, Rockville MD, 20850, USA; 8 Hudson Alpha Institute for Biotechnology, Huntsville AL, 35806, USA; 9 Department of Screening and Protein Sciences, Merck Research Laboratories, North Wales PA, 19454, USA; 10 Bristol-Myers Squibb Company, Applied Genomics, Princeton NJ, 08543, USA; 11 Seattle Genetics, Bothell WA, 98021, USA; 12 Division of Dermatology, Department of Medicine, University of Washington, Seattle WA, 98195, USA; 13 The Institute for Stem Cell and Regenerative Medicine, Seattle WA, 98109, USA

## Abstract

The inability of targeted BRAF inhibitors to produce long-lasting improvement in the clinical outcome of melanoma highlights a need to identify additional approaches to inhibit melanoma growth. Recent studies have shown that activation of the Wnt/β-catenin pathway decreases tumor growth and cooperates with ERK/MAPK pathway inhibitors to promote apoptosis in melanoma. Therefore, the identification of Wnt/β-catenin regulators may advance the development of new approaches to treat this disease. In order to move towards this goal we performed a large scale small-interfering RNA (siRNA) screen for regulators of β-catenin activated reporter activity in human HT1080 fibrosarcoma cells. Integrating large scale siRNA screen data with phosphoproteomic data and bioinformatics enrichment identified a protein, FAM129B, as a potential regulator of Wnt/β-catenin signaling.  Functionally, we demonstrated that siRNA-mediated knockdown of FAM129B in A375 and A2058 melanoma cell lines inhibits WNT3A-mediated activation of a β-catenin-responsive luciferase reporter and inhibits expression of the endogenous Wnt/β-catenin target gene, AXIN2. We also demonstrate that FAM129B knockdown inhibits apoptosis in melanoma cells treated with WNT3A. These experiments support a role for FAM129B in linking Wnt/β-catenin signaling to apoptosis in melanoma.

## Introduction

The incidence of melanoma continues to rise across the U.S. at a rate faster than any other cancer ^2^. Malignant melanoma has a poor prognosis with a 5-year survival rate of only 15% ^3^. The recently approved therapeutic, vemurafenib, extends median patient survival by 7 months ^4– 6^. This major advance raises expectations that even greater rates of survival might be attainable with combination therapies.

Activation of the Wnt/β-catenin pathway decreases tumor growth and cooperates with ERK/MAPK pathway inhibitors to promote apoptosis in melanoma ^1, 6– 12^. Analysis of melanoma tumor samples show a positive correlation between nuclear β-catenin staining and decreased tumor depth, increased patient survival and increased time to metastasis ^1, 7– 9^. Moreover, treatment with WNT3A-containing conditioned media or stable overexpression of WNT3A in mouse B16 or human A375 melanoma cells reduces cell number *in vitro* ^9– 11^. Allografts of mouse B16 or mouse xenografts of human A375 cells overexpressing WNT3A decrease tumor size compared to control ^9, 11^. Recently, we found that activation of Wnt/β-catenin signaling concurrent with the inhibition of the ERK/MAPK pathway synergistically elevates apoptosis in a subset of *BRAF*- and *NRAS*-mutant cultured human melanoma cells ^11, 12^. Given the interaction between Wnt/β-catenin signaling and pathways known to be critical for melanoma pathogenesis, the identification of Wnt/β-catenin regulators might prove to be informative in developing novel approaches to treat this disease.

In the present study, we identify novel regulators of Wnt/β-catenin signaling in melanoma by performing a large-scale small-interfering RNA (siRNA) screen of a Wnt/β-catenin responsive reporter in human HT1080 fibrosarcoma cells, and by identifying siRNA targets that are also regulated by ERK/MAPK signaling and that have been previously associated with melanoma. By integrating these three approaches, we identified FAM129B as a potential regulator of Wnt/β-catenin signaling. FAM129B is a 746 amino acid protein that contains an amino-terminal pleckstrin homology (PH) domain and a differentially phosphorylated carboxy-terminal region ^13^. FAM129B is known to inhibit TNFα-dependent apoptosis in HeLa cells ^14^. FAM129B is expressed in melanoma and promotes tumor cell invasion into collagen matrices in an ERK/MAPK phosphorylation-dependent manner ^13^. In the present study we demonstrate that FAM129B promotes Wnt/β-catenin signal transduction in melanoma cells and that reducing levels of FAM129B with siRNA reduces the ability of WNT3A to increase apoptosis in melanoma cells.

## Results

### Phosphoproteomic and siRNA screens identify FAM129B as a regulator of Wnt/β-catenin signaling

**Figure 1.**
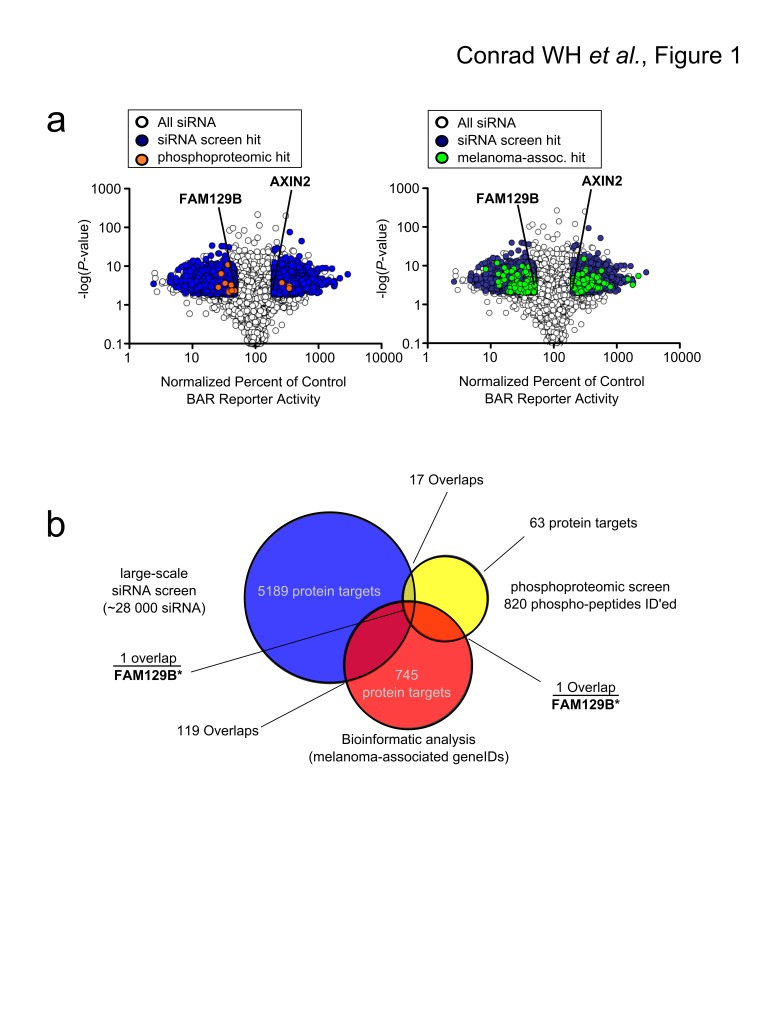
FAM129B is identified as a putative regulator of Wnt/β-catenin signaling using large-scale siRNA screen integrated with phosphoproteomic and bioinformatic analyses. ( **a**) Volcano plots depicting siRNA screen hits overlaid with phosphoproteomic data or bioinformatic data (left panel). Median effect of each siRNA treatment as a percent of control siRNAs were plotted against the p-value of that treatment. If, for a given gene, all siRNAs targeting that gene showed a twofold change in normalized reporter activity and a p-value <0.01, that gene was classified as a hit. This screen identified 5,189 gene products as hits, which are depicted in blue. Overlapping phosphoproteomic data from Old WM *et al.* (2009) ^7^ are depicted in orange. The known regulator of Wnt/β-catenin signaling, *AXIN2*, is indicated, as is FAM129B (right panel). Data plot is the same as the left panel with melanoma-associated genes plotted in green instead. ( **b**) Venn diagram depicting overlaps between phosphoproteomic dataset, siRNA screen and melanoma-associated genes. 17 protein targets overlap between the phosphoproteomic hits and the siRNA screen, 1 protein target overlaps between the phosphoproteomic hits and melanoma-associated protein targets, and 119 proteins overlap between the siRNA screen hits and melanoma associated protein targets. Only FAM129B overlaps with all three datasets.

In order to identify novel regulators of Wnt/β-catenin signaling, we performed a siRNA screen. We used HT1080 cells stably transduced with a luciferase reporter of β-catenin-mediated transcription (BAR) ^15^. We screened 28,044 pools of siRNAs. 19,490 gene products were targeted by one or more siRNA pool. Cells were transfected with siRNAs and treated with WNT3A-conditioned media to activate the reporter. BAR activity was normalized to the activity of *Renilla* luciferase driven by the constitutive TK promoter to control for total cell number. siRNAs targeting positive control proteins such as the known Wnt/β-catenin inhibitor, *AXIN2,* modulated BAR activity by at least 2.0 fold with a p-value less than 0.01 ( Figure 1b). Using this as a criterion, we found that 10,215 siRNA pools regulated BAR activity. Of the 19,490 gene products targeted by one or more siRNA in our screen, we identified 5189 gene products for which every given siRNA significantly regulated BAR activity ( Data File 1).

To refine the results of our large-scale siRNA screen, we performed an integrative analysis of our siRNA screen regulators by cross-referencing these regulators with a list of genes previously identified in melanoma, and a list of gene products phosphorylated downstream of MEK and ERK in melanoma. First, we identified 17 proteins in common between the siRNA and phosphoproteomic screens ( Figure 1a and Data File 3a). Next, we generated a list of melanoma-associated genes using a custom biopython script ( Data File 2 and Query Script). We identified 745 melanoma-associated genes by querying the NCBI gene database. Of these, one gene ( *FAM129B*) encoded a protein that was differentially phosphorylated following MEK inhibition ( Figure 1 and Data File 3a) and 119 were gene targets of siRNA pools that regulated Wnt/β-catenin signaling ( Figure 1b and Data File 3b). Finally, we discovered *FAM129B* as the only melanoma-associated gene that both modulated Wnt/β-catenin signaling and was phosphorylated following MEK activation, ( Figure 1b and Data File 3a).

UPDATED: Data sets and query script used in identifying FAM129B as a putative regulator of Wnt/β-catenin signaling using a largescale siRNA screen integrated with phosphoproteomic and bioinformatic analyses; and, FAM129B protein-protein interaction data. Results of affinity purification of Flag-GFP-FAM129B followed by mass spectrometric peptide identification. Column a, bait protein used for affinity purification. Column b, prey proteins identified by mass spectrometry. Column c, total peptides identified for a given prey peptide (spectral counts).

### Validation of FAM129B as a regulator of Wnt/β-catenin signaling

**Figure 2.**
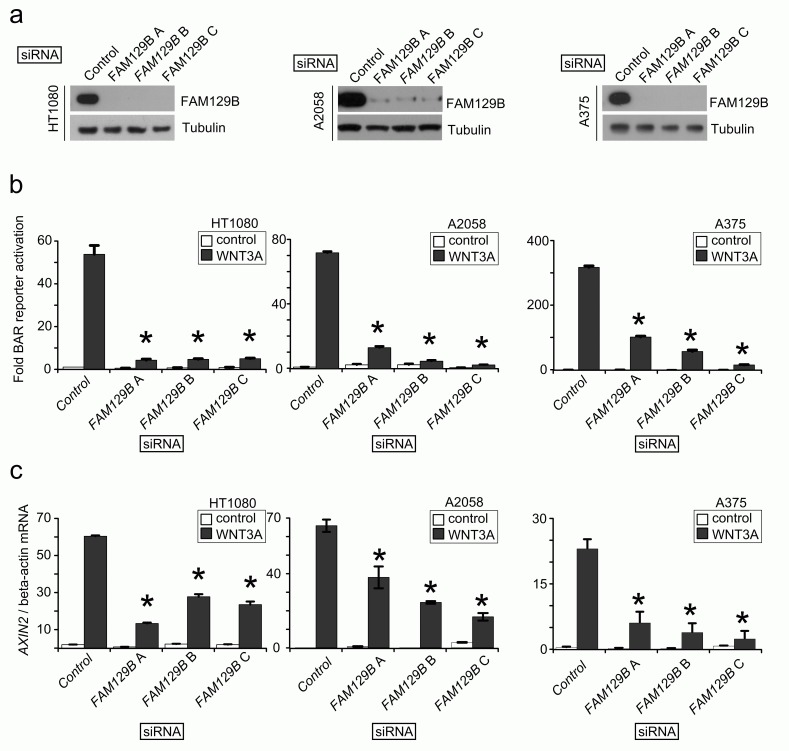
FAM129B positively regulates Wnt/β-catenin signal transduction in a panel of three cell lines. ( **a**) Immunoblots show three independent siRNAs reduce steady-state levels of endogenous FAM129B following 72 hr treatment with 20 nM siRNA. The beta-tubulin immunoblot serves as a control. Three independent siRNAs targeting *FAM129B* inhibit FAM129B expression in HT1080 (left), A2058 (middle), and A375 cells (right). ( **b**) *FAM129B* siRNA inhibit WNT3A-dependent luciferase reporter activity (BAR reporter) normalized to constitutively expressed *Renilla* luciferase in HT1080 (left), A2058 (middle), and A375 cells (right). ( **c**) *FAM129B* siRNA inhibit Wnt-dependent *AXIN2* expression in HT1080 (left), A2058 (middle), and A375 cells (right) relative to beta-actin mRNA expression by qPCR. Columns and error bars represent mean and SEM, respectively. Data are representative of at least three separate biological replicates. *p<0.05 by unpaired, two-tailed T-test.

The siRNA screen suggested that FAM129B is a regulator of Wnt/β-catenin signaling. In order to confirm this possibility, we designed three independent siRNAs targeting *FAM129B*. First, we confirmed that all three siRNAs inhibit expression of FAM129B protein in HT1080 fibrosarcoma, A2058 melanoma and A375 melanoma cells ( Figure 2a). Next, we asked whether the siRNAs inhibited the ability of WNT3A to activate BAR. Indeed, we found that each FAM129B siRNA reduced the ability of WNT3A to activate BAR in all three cell lines ( Figure 2b). We also tested whether FAM129B siRNAs reduce the ability of WNT3A to elevate expression of the endogenous β-catenin target gene, *AXIN2*. Similar to inhibition of BAR, *FAM129B* siRNAs significantly reduced levels of *AXIN2* transcript relative to control siRNA ( Figure 2c). From these data, we conclude that *FAM129B* knockdown inhibits the ability of WNT3A to promote β-catenin mediated transcriptional activation.

**Figure 3.**
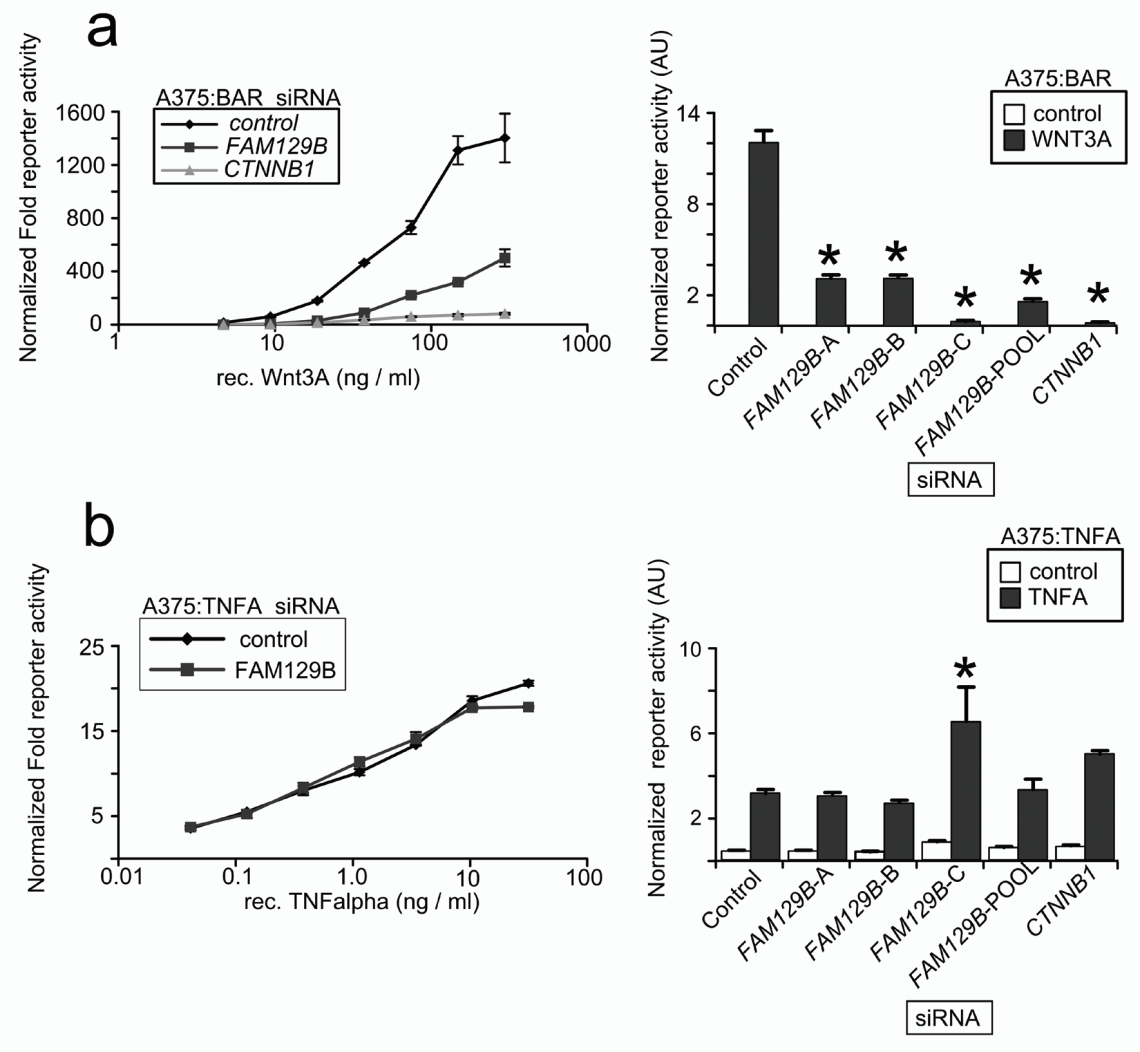
*FAM129B* siRNA regulate Wnt-dependent transcriptional reporter, but not TNFα/NFκB dependent reporter. ( **a**, left panel) pooled FAM129B siRNAs inhibit Wnt-dependent BAR reporter activity over a wide range of doses. Increasing doses of WNT3A increases activation of the BAR reporter (normalized to constitutive *Renilla* luciferase in control treated cells). WNT3A does not activate the reporter in the presence of FAM129B or *CTNNB1* siRNAs. (Right panel) A375 cells were treated with siRNAs as indicated and treated with an EC50 dose of WNT3A (50 ng/ml). All *FAM129B* siRNA and positive control *CTNNB1* siRNA inhibit Wnt-dependent BAR reporter activity. ( **b**) The same experiment was carried out as in ( **a**, left panel) in A375 lines TNFα/NFκB reporter. Data in the left panel indicate dose-dependent activation of the NFκB reporter by TNFα. However, *FAM129B* siRNAs do not inhibit the activation of the TNFα/NFκB reporter. (Right panel) *FAM129B* siRNA do not regulate activity of the NFκB reporter activated by 1.5 ng TNFα/ml in A375 cells. High dose TNFα (10 ng/ml) does differentially activate the reporter. Data represent 3 separate biological replicates. *p<0.05 by unpaired, two-tailed T-test.

While FAM129B modulates Wnt/β-catenin signaling in the above assays, these experiments do not rule out the formal possibility that reducing levels of FAM129B might affect other signaling pathways. We therefore generated A375 melanoma cell lines stably transduced with a luciferase-based reporter to the TNFα pathway. We then transfected cells with *FAM129B* siRNAs and stimulated the reporters with cognate ligands. While *FAM129B* siRNAs inhibit activation of the BAR reporter by WNT3A across a wide range of doses ( Figure 3a), *FAM129B* siRNA has only negligible effects on TNFα-dependent NFκB reporter activity ( Figure 3b). While this result does not allow the conclusion that FAM129B functions solely as a modulator of β-catenin signaling, this result does suggest that FAM129B is not required for activation of all pathways.

### FAM129B regulates WNT3A-mediated apoptosis in A375 melanoma cells

**Figure 4.**
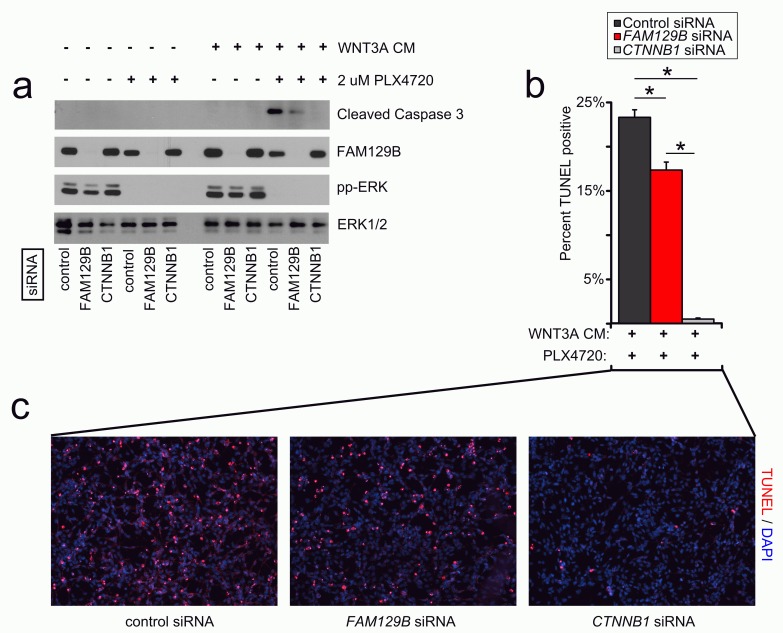
FAM129B positively regulates Wnt/β-catenin-dependent apoptosis in A375 melanoma. ( **a**) *FAM129B* siRNA inhibits Wnt-dependent apoptosis as monitored by cleaved caspase-3 immunoblot. A375 cells were treated with pooled control, pooled *FAM129B* siRNA, or *CTNNB1* siRNA as indicated for 48 hr. Cells were subsequently treated with DMSO or 2 µM PLX4720, and L-conditioned or WNT3A-conditioned media for 24 hr as indicated. Knockdown of FAM129B was monitored by FAM129B immunoblot, inhibition of ERK/MAPK signaling by phospho-ERK immunoblot, and total ERK was used as normalization. Relative levels of cleaved caspase-3 were quantitated by normalizing cleaved caspase-3 pixel density to ERK1/2 for each condition relative to the maximum cleaved caspase-3 level. Data are representative of at least 3 biological replicates. *FAM129B* siRNA inhibit cleaved caspase-3 levels to between 16 and 41% of maximum. ( **b**) *FAM129B* siRNA inhibits Wnt-dependent apoptosis as quantified by terminal deoxynucleotidyl transferase-mediated deoxyuridine triphosphate nick end labeling (TUNEL) immunofluorescence (IF). A375 melanoma cells were treated as above, fixed and stained using TUNEL. Percent TUNEL positive cells calculated as a percent of DAPI positive cells. ( **c**) Representative immunofluorescence of A375 cells treated with the indicated conditions. TUNEL staining is depicted in red and DAPI staining is depicted in blue. Columns and error bars represent the mean and SEM of three separate biological replicates. *p<0.05 by student’s T-test.

The combined treatment with WNT3A protein and compounds that inhibit ERK/MAPK signaling synergizes to induce robust apoptosis in cultured melanoma cells ^11, 12^. If FAM129B is required for Wnt/β-catenin signaling, then FAM129B loss of function should inhibit this synergy. We monitored apoptosis in A375 melanoma cells by western blot for cleaved caspase-3 and immunofluorescence staining for TUNEL (terminal deoxynucleotidyl transferase-mediated deoxyuridine triphosphate nick end labeling). As previously reported ^11, 12^, A375 cells treated with control siRNA and the combination of WNT3A and PLX4720 exhibit robust levels of cleaved caspase-3 ( Figure 4a). siRNA mediated knockdown of *FAM129B* decreases the levels of cleaved caspase-3 in response to WNT3A siRNA ( Figure 4a–4c). Moreover, when measuring WNT and PLX4720-dependent apoptosis by TUNEL staining, we found that siRNA mediated FAM129B knockdown reduced the number of TUNEL positive cells as compared to control siRNAs. Collectively, these results show that FAM129B is required for the synergy between Wnt3A and PLX4720 to induce melanoma apoptosis.

### FAM129B expression is elevated in human melanoma cohorts with increased invasiveness and decreased activation of Wnt/β-catenin signaling

**Figure 5.**
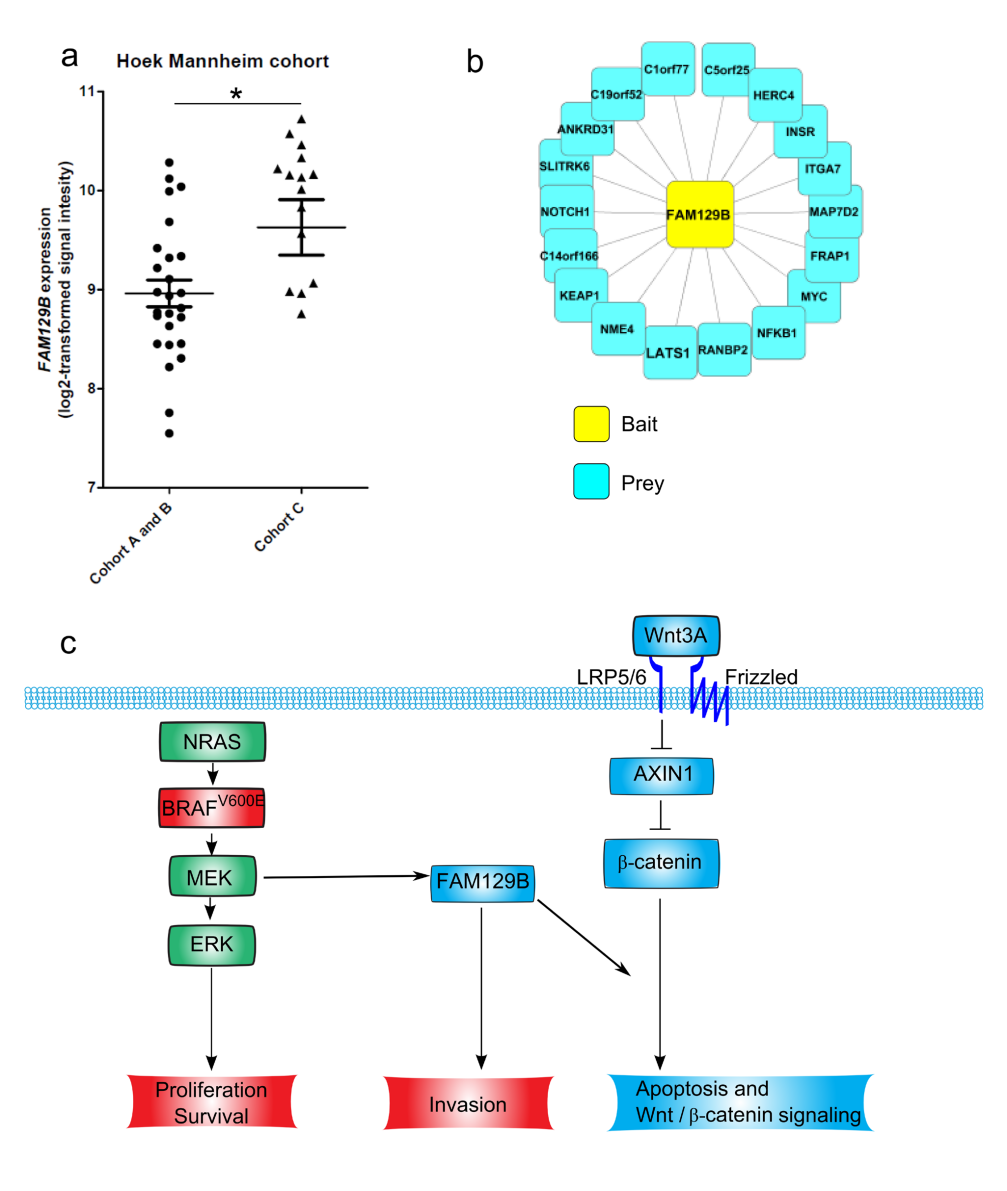
( **a**) FAM129B expression associates with an invasive cohort of patient melanomas. *FAM129B *gene expression was compared between three previously identified melanoma cohorts. We compared *FAM129B *expression in cohorts A and B with cohort C. Log(2) transformed expression for FAM129B was significantly higher in cohort C than in cohorts A and B (8.962 ± 0.1355 vs 9.629 ± 0.2795, p= 0.0214) ( **b**) FLAG-purification of FLAG-GFP-FAM129B unveils multiple FAM129B interactors. Nodes depict FAM129B interacting proteins identified by affinity purification/mass spectrometry. Bait protein Flag-GFP-FAM129B is depicted in yellow. Prey proteins are depicted in blue. ( **c**) Model of FAM129B regulation by ERK/MAPK signaling in melanoma and regulation of Wnt/β-catenin signaling. FAM129B is a substrate downstream of active MEK (green) that is required for melanoma invasion ^13^. We identified FAM129B as a positive regulator of Wnt/β-catenin signaling (blue).

Given that *FAM129B* silencing inhibits Wnt/β-catenin target gene expression and apoptotic response to WNT3A, we sought to determine if *FAM129B* expression levels predict Wnt/β-catenin pathway activation in patient melanoma samples. We analyzed published microarray data from Hoek *et al.* ^16^. These authors identify three patient cohorts using unsupervised hierarchical clustering on gene expression data from multiple datasets ^16^. Cohorts A and B have a high Wnt/β-catenin/ *MITF* signature and were designated "proliferative" cohorts. Cohort C has very high *WNT5A* and was designated the "invasive" cohort ^16^. We observe that *FAM129B* expression is significantly higher in cohort C than in cohorts A and B ( Figure 5a). These data indicate that *FAM129B* expression associates with an invasive phenotype, rather than with elevated Wnt/β-catenin signaling in melanoma patients. Consistent with this observation, we did not observe a direct correlation between levels of *FAM129B* and expression of the Wnt/β-catenin target gene *AXIN2* (data not shown) in these three cohorts.

### FAM129B interacting proteins identified by affinity purification/mass spectrometry

FAM129B has no known enzymatic domains and therefore likely exerts its cellular effects via protein-protein interactions. In the interest of identifying the mechanism of regulation of Wnt/β-catenin signaling by FAM129B, we identified FAM129B interacting proteins by affinity purifying an N-terminal Flag- and GFP-tagged FAM129B protein and its interactors from A375 melanoma cells. We subsequently identified interacting proteins by mass spectrometry (see materials and methods). We identified 18 FAM129B interacting proteins, including the previously identified FAM129B interacting protein KEAP1 ( Figure 5b and Data File 4) ^17^. Our preliminary research validating these FAM129B protein-protein interactions has not yet identified the interaction responsible for regulation of Wnt/β-catenin signaling. These protein-protein interaction data are provided for future research into FAM129B cellular function.

Data showing positive regulation of Wnt/β-catenin signal transduction by FAM129B siRNA using transcriptional reporter assay, target gene expression, and apoptosis assay Data for Figure 2b. Measured β-catenin dependent firefly: constitutive renilla ratios for HT1080, A375, and A2058 reporter cells treated with the indicated conditions. Data for Figure 2c: Quantitative RT-PCR of β-catenin dependent Axin2 mRNA and housekeeping actin mRNA for HT1080, A375, and A2058 cells treated with the indicated conditions. Data for Figure 3a. Measured β-catenin dependent firefly: constitutive renilla ratios for A375 reporter cells treated with a dose curve of WNT3A or a single dose of WNT3A. Data for Figure 3b. Measured NFκB dependent firefly: constitutive renilla ratios for A375 reporter cells treated with a dose curve of TNFalpha or a single dose of TNFα . Data for Figure 4b. Quantitation of DAPI and TUNEL positive nuclei from A375 cells treated with WNT3A, PLX4720, and the control, FAM129B, or CTNNB1 siRNA.

## Discussion

We combined phosphoprotoemics and siRNA screening to identify novel regulators of Wnt/β-catenin signaling in human melanoma. We focused on FAM129B, a previously identified protein that has not formerly been linked to Wnt/β-catenin signaling. Using independent siRNAs, we confirmed that *FAM129B* is required for Wnt3A to activate a β-catenin dependent reporter and reduces the ability of Wnt3A to enhance the expression of the β-catenin target gene *AXIN2*. We demonstrated that loss of function of FAM129B inhibits the apoptosis of melanoma cells induced by the combined treatment with WNT3A and PLX4720.

Elevated Wnt/β-catenin signaling predicts improved prognosis in melanoma patients ^9^, slows the growth of melanoma xenografts *in vivo*, and cooperates with inhibition of the ERK/MAPK pathway to promote apoptosis of melanoma cells *in vitro* ^9, 11, 12^. There is accumulating data suggesting that Wnt/β-catenin signaling is a key regulator of melanoma metastasis. Wnt/β-catenin signaling has been implicated as the key phenotypic switch that can regulate whether melanoma cells exhibit either a proliferative or an invasive phenotype ^8, 18^. In mouse models of melanoma, Wnt/β-catenin signaling was recently identified as a key regulator of the metastatic phenotype ^18^. Interestingly, FAM129B was originally identified as a protein that promotes the invasion of melanoma cells following regulated phosphorylation by the MAPK pathway ^13^, and our own analysis showing an increased expression of FAM129B in more “invasive” melanoma cohorts supports the important role of FAM129B in melanoma suggested by this initial observation ( Figure 5a). Our identification of FAM129B as regulator of the Wnt/β-catenin pathway places FAM129B at a node of cross-talk between two signaling pathway simplicated in the regulation of melanoma pathogenesis ( Figure 5c).

One obvious conundrum is the observation that *FAM129B* expression is relatively lower in cohorts exhibiting higher levels of Wnt/β-catenin activation. Based on our observations that FAM129B can positively regulate apoptosis in cells with high levels of Wnt/β-catenin signaling, it is conceivable that cells expressing high levels of FAM129B within the cohorts with high Wnt/β-catenin signaling may preferentially undergo apoptosis, thus favoring the survival of cells with low levels of FAM129B. Although this hypothesis would be extremely difficult to verify, it could plausibly account for the relatively lower levels of FAM129B seen in cohorts with increased Wnt/β-catenin signaling. Another distinct possibility is that the role of FAM129B as a MAPK-dependent regulator of cellular invasion may supersede its role as a regulator of Wnt/β-catenin signaling in the *in vivo* tumor environment.

The predicted role of FAM129B as an intracellular scaffolding protein suggests that direct therapeutic targeting of the protein itself would be difficult if not impracticable. However, the previously identified regulation of FAM129B by BRAF/MAPK signaling is intriguing given the observation that enhanced Wnt/β-catenin signaling can augment apoptosis with targeted BRAF inhibitors, which are currently first-line therapy in metastatic melanoma patients whose tumors harbor activating mutations in BRAF. Whether FAM129B or other regulators of Wnt/β-catenin signaling may determine the variability in clinical response or the eventual acquisition of resistance seen in patients treated with this class of drugs remains to be seen. Further studies identifying additional mechanisms that regulate both FAM129B and Wnt/β-catenin signaling in melanoma cells will undoubtedly clarify whether this interaction has any significance as a prognostic biomarker or as a downstream target for pathway-based melanoma therapies including targeted BRAF inhibitors.

*FAM129B* siRNAs suppress apoptosis in melanoma cells treated with WNT3A and PLX4720. Knockdown of FAM129B suppresses apoptosis to a lesser extent than Wnt/β-catenin signaling likely because Wnt/β-catenin signaling synergistically increases apoptosis in combination with BRAF inhibitors ^11^. An isobologram analysis of WNT3A and the BRAF-inhibitor PLX4720 revealed a synergistic inhibition of viability between WNT3A and BRAF inhibition ^11^. Thus, even low level activation of Wnt/β-catenin signaling in the absence of FAM129B can still potently promote apoptosis in combination with BRAF inhibition. Nonetheless, this result was surprising given that the transfection of *FAM129B* siRNA in HeLa cells promotes increased apoptosis in response to TNFα and cyclohexamide ^14^. The discrepancy between the ability of *FAM129B* siRNAs to suppress Wnt-dependent apoptosis in melanoma and the ability of these siRNA to promote TNFα-mediated apoptosis in HeLa remains unresolved, although it does suggest that FAM129B may function in a manner that is dependent on cellular context. Alternatively, the differences in apoptotic response with FAM129B loss of function may merely reflect the regulation of Wnt/β-catenin signaling in these two cell types. Uncovering the underlying roles of FAM129B in the cell may well illuminate how FAM129B exerts these opposing effects on apoptosis in response to different stimuli. Future studies should probe the role, if any, of TNFα/NFκB in melanoma apoptosis and the cross-talk between Wnt/β-catenin and TNFα/NFκB signaling in cell lines, such as HeLa, that respond to TNFα by apoptosis.

## Materials and methods

### Plasmids

Detailed information on the β-catenin activated reporter plasmid (pBARLS) has been previously described ^15, 19^. Briefly, the reporters are generated from lentiviral plasmids that contain 12 TCF/LEF binding sites (5´-AGATCAAAGG-3´) or Nuclear Factor Kappa B (5´-GGGAATTTCC-3´) signaling pathways separated by distinct 5-base pair linkers upstream of a minimal promoter and the firefly luciferase open reading frame. The reporters also contain a separate PGK (phosphoglycerate kinase) promoter that constitutively drives the expression of a puromycin resistance gene for mammalian cell selection. These reporters were generated by Travis L. Biechele in the lab of Randall T Moon as previously published ^15, 19^.

### Cell lines and cell culture

Human A375 and A2058 cells were a generous gift from Cassian Yee (Fred Hutchinson Cancer Research Institute, Seattle, WA). HT1080 cell lines were purchased from the American Type Culture Collection (ATCC, Manassas, VA). Stable reporter lines were generated as previously described ^15^. Cell lines were cultured in a Thermo Forma steri-cult humidified incubator (#3310, Thermo Scientific, Rockford, IL) at 37°C and 5% CO _2_. All cell lines were cultured in Dulbecco’s Modified Eagle’s Medium (DMEM, #11965–084 Invitrogen, St. Louis, MO) containing 10% fetal bovine serum and 1% Penicillin/Streptomycin (Invitrogen, Grand Island, NY), except A375 cells, which were grown in DMEM containing 5% FBS and 1% P/S.

Control (LCM) and WNT3A-conditioned media (WNT3A CM) used to activate the Wnt/β-catenin signaling pathway were prepared as previously described ^20^. To monitor reporter activity and transcript activity, cells were treated with 10% WNT3A CM or LCM overnight before proceeding to subsequent assays. To monitor effects on apoptosis, cells were treated with 1% LCM or WNT3A and DMSO (Sigma St. Louis, MO, product 472301) or 2 uM PLX4720 (Symansis, Timaru New Zealand SY-PLX4720).

### Large-scale siRNA screen

The large-scale siRNA screen was performed as previously described ^21^, with minor modifications. Briefly, HT1080 cells stably transduced with BAR firefly luciferase and *Renilla* luciferase lentivirus were reverse-transfected in 1536-well plates, with a final concentration of pooled siRNA at 25 nM. 48 hours after reverse transfection, cells were treated with WNT3A-conditioned media. Following overnight incubation, β-catenin dependent transcription was measured by assaying firefly luciferase activity and normalized by monitoring constitutively expressed *Renilla* luciferase activity as described in the Promega Dual glo luciferase assay system technical manual (Promega, Madison Wi). All siRNAs were designed with a proprietary algorithm ^22^.

### siRNA transfection and low throughput reporter assays

Approximately 200,000 A375, A2058, or HT1080 cells (as estimated by hemocytometer counts) were reverse transfected at a final dose of 20 nM siRNA in 6-well format using 5 µl RNAi max/well (Invitrogen, Grand Island, NY). Medium GC universal stealth control siRNA was used as a negative control (Cat. No. 12935–112, Invitrogen, Grand Island, NY). Invitrogen’s stealth siRNA targeting *FAM129B* were designed using the BLOCK-iT RNAi designer and are described below. The sequence for "FAM129B A" is UCACGGACAUGAACCUGAACGUCAU. The sequence for "FAM129B B" is ACUGAGGUGCGAGAUGUCUUCUUCA. The sequence for "FAM129B C" is CAGCAGCGAUUUGAUGUGUCCAGCA. As a positive control for inhibition of Wnt/β-catenin signal transduction by siRNA, we used silencer select siRNA targeting *CTNNB1* with the sequence GGUGGUGGUUAAUAAGGCUTT (Invitrogen, Grand Island, NY).

24 hr after siRNA transfection, cells were plated in 96-well plates at a density of 20,000 cells/well. Twenty-four hours after plating, cells were treated with the indicated conditions, and luciferase activity was measured 15 hours later with a Dual-Luciferase Reporter Assay kit (Promega, Madison, Wi) and an Envision multilabel plate reader (PerkinElmer, Waltham, MA) according to the manufacturer’s suggestions.

### qPCR

24 hr after siRNA transfection, cells were split into a 12-well cluster plate at approximately 50% confluency. 24 hr later, cells were treated with WNT3A- or L-conditioned media. After overnight treatment, RNA was isolated using Trizol reagent according to the manufacturer’s instructions (Invitrogen). 1 µg of RNA was reverse transcribed using Fermentas’ RevertAid M-MuLV Reverse Transcriptase (Fermentas, Glen Burnie, MD). QPCR was performed on a Lightcycler 480 (Roche, Indianapolis, IN) using Lightcycler 480 DNA SYBR Green 1 master mix (04707516001 Roche, Indianapolis, IN). The following primers were used for qPCR: " *AXIN2* F" CTCCCCACCTTGAATGAAGA and " *AXIN2* R" TGGCTGGTGCAAAGACATAG; and, " *ACTB* F" AGAGCAAGAGAGGCATCCTC and " *ACTB* R" CTCAAACATGATCTGGGTCA.

### Cell lysis and immunoblotting

To test for siRNA knockdown, replicate cell lysates from low throughput reporter assays were pooled and treated with 10× RIPA lysis buffer (500 mM Tris, pH 7.5, 1.5 M NaCl, 10 mM EDTA, 10% Igepal CA-630, 1% SDS, and 2% sodium deoxycholate all purchased from Sigma, St. Louis, MO). For monitoring cleaved caspase-3, 90% confluent 12-well plates were treated for 24-hr with the indicated conditions described in the "cell lines and cell culture" section. Media were collected and cells were rinsed once (gently) with PBS. Cells were lysed on-plate in 100 µl 1× RIPA buffer containing protease and phosphatase inhibitors (Complete EDTA-free and PhoStop by Roche, Indianapolis, IN). Cells were disrupted by scraping of a 1000 µl pipette tip against the plate. Apoptotic cells present in the media and PBS wash were centrifuged at 300 *g*, rinsed once with PBS, and lysed with the RIPA buffer collected from the plate lysis. Cell lysates were cleared by centrifugation at 20,000 *g* at 4°C for 10 minutes. Protein lysates were separated by SDS-PAGE using NuPAGE 4%–12% Bis-Tris gels (NP0336BOX, Invitrogen, Grand Island, NY) in MES buffer, and transferred onto a nitrocellulose membrane (162–0115, Bio-Rad, Hercules, CA) using IDEA scientific GENIE transfer apparatuses (Idea Scientific, Minneapolis, MN). Blots were probed using polyclonal rabbit anti FAM129B (#HPA023261 Sigma, St. Louis, MO), monoclonal mouse anti Tubulin (#T7816 Sigma, St. Louis, MO), monoclonal mouse anti β-catenin (C2206 Sigma, St. Louis, MO), polyclonal Rabbit anti cleaved-caspase-3 (#9661 cell signaling), Rabbit anti ERK1/2 (#9102 cell signaling, Danvers, MA), Rabbit anti phospho ERK1/2 (#9211 cell signaling, Danvers, MA).

### TUNEL immunofluorescence

Glass coverslips were coated with poly- l-lysine in a 24-well dish, rinsed with PBS, and dried. Following reverse transfection as described above, cells were seeded at a density to achieve 90 to 100% confluency at harvest. Twenty-four hours after seeding, cells were treated with the indicated conditions and incubated for 24 hours with the indicated conditions as described above in the "cell lines and cell culture" section. Terminal deoxynucleotidyl transferase dUTP nick end labeling (TUNEL) staining was performed using an *in situ* cell death detection kit (Roche, Indianapolis, IN). Briefly, the medium was gently aspirated, to keep apoptotic bodies on the slide, and cells were fixed in 4% paraformaldehyde for 1 hour at room temperature. Cells were gently rinsed twice with PBS and permeabilized with 0.1% Triton X-100 (Sigma, St. Louis, MO) in 0.1% sodium citrate (Sigma, St. Louis, MO) for 2 min on ice. Cells were rinsed twice with PBS and 40 ml of TUNEL reaction mixture was added directly on top of the slide; cells were incubated for 1 hour at 37°C in a humidified incubator. Slips were rinsed three times with PBS and mounted on Superfrost Plus glass slides with ProLong Gold anti-fade mounting medium containing 4´,6-diamidino-2-phenylindole (DAPI) (Invitrogen, Grand Island, NY). Images were obtained on a Nikon TiE inverted wide-field high-resolution microscope. DAPI and TUNEL positive nuclei were quantified blinded for 5 fields per slide using NIS elements (Nikon Instruments Inc, Melville, NY).

### Statistics

Except where indicated, a student’s t test was used to assess the statistical significance of the differences between the different groups; a p value of <0.05 was considered significant.

### Affinity purification and mass spectrometry

Flag-GFP-FAM129B and associated proteins were isolated from A375 cells as described previously ^23, 24^. Briefly, cleared protein extracts generated from 1 × 10 ^9^ A375 cells stably transduced Flag-GFP-FAM129B were incubated with 20 μl Anti-Flag M2 affinity gel (A2220, Sigma, St. Louis, MO) at 4°C for 4 hr. After extensive washing, interacting affinity gel was treated with sequencing grade trypsin (V5113, Promega, Madison, Wi) and analyzed by tandem mass spectrometry as described previously ^23^. Interacting proteins also copurified by a Flag-GFP control or commonly copurified by unrelated Flag-affinity purifications were subtracted from the final protein-protein interaction list.
