# Supplementary material for: FAM129B is a novel regulator of Wnt/β-catenin signal transduction in melanoma cells
Source: F1000Res. 2013 Oct 10;2:134. Originally published 2013 May 31. [Version 2] doi: 10.12688/f1000research.2-134.v2 (PMC3829391; doi:10.12688/f1000research.2-134.v2)
Supplement: UPDATED: Data sets and query script used in identifying FAM129B as a putative regulator of Wnt/β-catenin signaling using a largescale siRNA screen integrated with phosphoproteomic and bioinformatic analyses; and, FAM129B protein-protein interaction data. — Results of affinity purification of Flag-GFP-FAM129B followed by mass spectrometric peptide identification. Column a, bait protein used for affinity purification. Column b, prey proteins identified by mass spectrometry. Column c, total peptides identified for a given prey peptide (spectral counts). [file f1000research-2-2455-s0001.tgz › Query_script.docx]

#Supplementary text 1 – Python script for listing disease-associated genes

#Requires installation of Python v2.6 or later, and Biopython v1.55 or later

#Also requires an internet connection, or local version of the NCBI gene database

import sys # import system functions from python

from Bio import Entrez # import Entrez search functions from biopython

Entrez.email = "wconrad@u.washington.edu" # Always tell NCBI who you are when using esearch

disease = sys.argv[1] # The user enters their disease term of interest

term1=disease+'[All Fields] AND (alive[PROP] AND "Homo sapiens"[Organism])'

# This is the term that entrez gene will search

handle = Entrez.esearch(db="gene", term=term1, retmax=800) # the biopython esearch for the above term is stored as the variable “handle”

record = Entrez.read(handle) #the record created by the term search is stored as "record"

hitlist = record["IdList"] #the hitlist variable collects all of the geneIDs from the original search

newfile = open(disease+"_genes.txt", "w") # a new tab-delimited text file is created that uses the search query as the title and also the word genes.

for id in hitlist:

newfile.write(disease+"\t"+"lit"+"\t"+str(id)+"\n") # this loop enters the query, the designation lit for 'literature interaction' and the geneID as as string.

newfile.close()

#print len(hitlist) #python displays the number of hits
