# Supplementary material for: FAM129B is a novel regulator of Wnt/β-catenin signal transduction in melanoma cells
Source: F1000Res. 2013 Oct 10;2:134. Originally published 2013 May 31. [Version 2] doi: 10.12688/f1000research.2-134.v2 (PMC3829391; doi:10.12688/f1000research.2-134.v2)
Supplement: Peer review history

# Peer review: Referee response for version 2

**Published:** 2013-10-31

**Authors:** Madelon Maurice

**Affiliations:** 1 Department of Cell Biology, University Medical Center Utrecht, Utrecht, Netherlands

I am satisfied with the changes made by the author(s).

I have read this submission. I believe that I have an appropriate level of expertise to confirm that it is of an acceptable scientific standard.

# Peer review: Referee response for version 2

**Published:** 2013-10-24

**Authors:** Uyadan Apte

**Affiliations:** 1 Department of Pharmacology, Toxicology and Therapeutics, University of Kansas Medical Center, Kansas City, KS, USA

I confirm that the manuscript is now approved.

I have read this submission. I believe that I have an appropriate level of expertise to confirm that it is of an acceptable scientific standard.

# Peer review: Referee response for version 1

**Published:** 2013-07-24

**Authors:** Uyadan Apte

**Affiliations:** 1 Department of Pharmacology, Toxicology and Therapeutics, University of Kansas Medical Center, Kansas City, KS, USA

1. The title is appropriate for the study.

2. The experimental design and methods are appropriate for the topic being studied.

3. The information and data provided is enough for replicating the experiment

4. The conclusions are correct but need additional data to substantiate it. In this article, the authors have identified FAM129B as a positive regulator of Wnt/beta-catenin signalling in melanoma cells. They also demonstrate that FAM129B is required for Wnt3a-induced apoptosis *in vitro*.

However; there are three important pieces of information missing that the authors should address:

- Can this effect be replicated *in vivo* in animal models?
- Does FAM129B expression correlate with beta-catenin activation and disease progression in melanoma patients?
- What is the mechanism by which FAM129B regulates Wnt signalling?

I would like the authors to do a bit more work on answering at least one of the three questions raised. They should also discuss the other two questions in the discussion section. From my standpoint it seems the easiest thing to do would be to compare FAM129 expression in various tumor samples and correlate them to Beta-catenin activation (nuclear localization) and growth pattern (tumor depth).

I have read this submission. I believe that I have an appropriate level of expertise to confirm that it is of an acceptable scientific standard, however I have significant reservations, as outlined above.

# Response

**Published:** 2013-10-10

**Authors:** William Conrad

**Affiliations:** University of Washington, Seattle, USA

Dear Dr. Apte,

We appreciate your thorough reading of our manuscript, as well as the thoughtful questions that you have raised in your review.  We have outlined a response to specific questions below:

1. *“Does FAM129B expression correlate with beta-catenin activation and disease progression in melanoma patients?” *
  This is a very interesting question, and one which we had sought to address using previously published data from defined cohorts of melanoma patients. We analyzed the abundance of FAM129B present in three different cohorts of melanoma (Figure 5a in version 2) and observed a significantly higher abundance of FAM129B mRNA in a melanoma cohort associated with higher invasiveness and relatively lower Wnt/β-catenin signaling. From this observation, we concluded that FAM129B abundance did not predict Wnt/β-catenin signaling in melanoma patients. This conclusion was supported by a lack of significant correlation between levels of *FAM129B* and expression of the established Wnt/β-catenin target gene, *AXIN2*, within this cohort (data not shown). We have included a brief discussion of these results in our revision in order to clarify the fact that we do not have data that supports a clear role of direct regulation of Wnt/β-catenin signaling by FAM129B in melanoma patients.
   
2. *“Can this effect be replicated in vivo in animal models?” *
  We did attempt to address this question, but despite repeated attempts we were unable to stably knock down FAM129B using lentiviral shRNA in melanoma cells.  As a result, we were not able to generate cell lines that could be used to address this question in murine xenografts.
   
3. *“What is the mechanism by which FAM129B regulates Wnt/β-catenin signaling?” *
  Unfortunately, we have been unable to solve the specific mechanism by which FAM129B regulates Wnt/β-catenin signaling.  We had hoped that a proteomics-based approach identifying proteins that interact with FAM129B might prove informative, but to date we have not solidified any mechanistic links based on the hits identified with this strategy.  While our efforts to validate this network are currently too preliminary for publication, we have (in the spirit of open access) included our FAM129B protein interaction network in its entirety in order to allow other readers to potentially gain insight and generate hypotheses regarding the role of this protein in regulating cellular signaling (Figure 5b). 

# Peer review: Referee response for version 1

**Published:** 2013-07-19

**Authors:** Madelon Maurice

**Affiliations:** 1 Department of Cell Biology, University Medical Center Utrecht, Utrecht, Netherlands

This report describes the identification of FAM129B as a novel regulator of Wnt/β-catenin signalling and apoptosis in melanoma cells. The FAM129B gene was identified via integration of 3 datasets, including Wnt signaling modifiers, melanoma-associated genes and the MEK/ERK phosphoproteome. FAM129B was the only gene that overlapped with all three datasets, making it a promising candidate for regulation of both Wnt and MEK/ERK pathways in melanoma. I find this an interesting report which will provide a good starting point for further studies. Overall, the data are convincing and support the conclusions drawn. Experiments which validate FAM129B as a Wnt pathway modifier (Fig. 1-3) appear solid and display robust effects. The effects of FAM129B on apoptosis (Fig. 4b), however, are somewhat lower than expected based on the effects of FAM129B knockdown in reporter assays and on the inhibition of caspase cleavage. I feel that the authors could provide a more in-depth discussion on their findings and place them in the context of the background information provided in the introduction part; 

How do the authors consider this candidate relevant for melanoma treatment? 

How do the authors integrate the beneficial effects of enhanced Wnt responsiveness (this report) and the previously described enhanced tumour cell invasion (ref 20) of FAM129B expressing melanoma cells?

It would be helpful for the reader to include a model which shows how Wnt and ERK pathways modulate apoptosis in melanoma cells and where FAM129B would be placed in these events. Also in  the legend of Fig. 2a it remains unclear whether endogenous levels of FAM129B are depicted.

I have read this submission. I believe that I have an appropriate level of expertise to confirm that it is of an acceptable scientific standard.

# Response

**Published:** 2013-10-10

**Authors:** William Conrad

**Affiliations:** University of Washington, Seattle, USA

Dear Dr. Maurice,

We appreciate your thorough reading of our manuscript, as well as the thoughtful questions that you have raised in your review.  We have outlined a response to specific questions below:

1. *“How do the authors consider this candidate relevant for melanoma treatment?”*
  It is unclear how FAM129B is relevant for melanoma treatment at this point.  Our data identifies FAM129B as a regulator of Wnt/β-catenin dependent phenotypes in melanoma cells, while previous studies had already identified FAM129B as a downstream protein regulated by ERK/MAPK signaling.  Given the importance of both of these signaling pathways in melanoma biology, we hypothesize that FAM129B could be an important regulatory nexus between Wnt/β-catenin and ERK/MAPK signaling, although at this point we have been unable to draw a direct connection in patient tumors. Thus, at this point it is difficult to make any conclusions on the role of FAM129B’s role in melanoma treatment without being overly speculative.
   
2. *“I feel that the authors could provide a more in-depth discussion on their findings and place them in the context of the background information provided in the introduction part”*
  We have added some more content to the discussion, and we hope that these changes have improved the manuscript.
   
3. *“How do the authors integrate the beneficial effects of enhanced Wnt responsiveness (this report) and the previously described enhanced tumour cell invasion (ref 20) of FAM129B expressing melanoma cells?” *
  You have raised a very interesting and relevant point regarding the difficulties in broadly generalizing results obtained in cultured cell systems across a disease. Our evaluation of published data (presented in this revision) also shows increased expression of FAM129B in a cohort previously defined as more invasive, and with lower activation of Wnt/β-catenin signaling (Figure 5a). These data would suggest that in patients, the effect of FAM129B on melanoma cell invasion may supersede any effects on regulating Wnt/β-catenin signaling.
   
4. In response to your helpful suggestion, we have added a model which shows how Wnt/β-catenin and ERK/MAPK pathways modulate apoptosis in melanoma cells (Figure 5c). 
   
5. Thank you for your careful reading of the figure legends, we have edited the legend of Figure 2a to clarify that our western blot of FAM129B is for endogenous FAM129B protein levels.
